# *AEBP1* expression increases with severity of fibrosis in NASH and is regulated by glucose, palmitate, and miR-372-3p

**DOI:** 10.1371/journal.pone.0219764

**Published:** 2019-07-12

**Authors:** Glenn S. Gerhard, Amanda Hanson, Danielle Wilhelmsen, Ignazio S. Piras, Christopher D. Still, Xin Chu, Anthony T. Petrick, Johanna K. DiStefano

**Affiliations:** 1 Lewis Katz School of Medicine, Temple University School of Medicine, Philadelphia, PA, United States of America; 2 Diabetes and Fibrotic Disease Unit, Translational Genomics Research Institute, Phoenix, AZ, United States of America; 3 Geisinger Obesity Institute, Danville, PA, United States of America; Vrije Universiteit Brussel, BELGIUM

## Abstract

Factors governing the development of liver fibrosis in nonalcoholic steatohepatitis (NASH) are only partially understood. We recently identified adipocyte enhancer binding protein 1 (*AEBP1*) as a member of a core set of dysregulated fibrosis-specific genes in human NASH. Here we sought to investigate the relationship between *AEBP1* and hepatic fibrosis. We confirmed that hepatic *AEBP1* expression is elevated in fibrosis compared to lobular inflammation, steatosis, and normal liver, and increases with worsening fibrosis in NASH patients. *AEBP1* expression was upregulated 5.8-fold in activated hepatic stellate cells and downregulated during chemical and contact induction of biological quiescence. In LX-2 and HepG2 cells treated with high glucose (25 mM), *AEBP1* expression increased over 7-fold compared to normal glucose conditions. In response to treatment with either fructose or palmitate, *AEBP1* expression in primary human hepatocytes increased 2.4-fold or 9.6-fold, but was upregulated 55.8-fold in the presence of fructose and palmitate together. *AEBP1* knockdown resulted in decreased expression of nine genes previously identified to be part of a predicted *AEBP1*-associated NASH co-regulatory network and confirmed to be upregulated in fibrotic tissue. We identified binding sites for two miRNAs known to be downregulated in NASH fibrosis, miR-372-3p and miR-373-3p in the *AEBP1* 3’ untranslated region. Both miRNAs functionally interacted with *AEBP1* to regulate its expression. These findings indicate a novel *AEBP1*-mediated pathway in the pathogenesis of hepatic fibrosis in NASH.

## Introduction

Nonalcoholic fatty liver disease (NAFLD) is a chronic, frequently progressive condition resulting from excessive accumulation of fat in hepatocytes. Nonalcoholic steatohepatitis (NASH) is a clinically advanced form of NAFLD characterized by hepatic inflammation with or without scarring that is associated with increased liver-related morbidity and mortality [1]. Severe liver fibrosis represents the end-stage pathology evolving from a number of pathogenic mechanisms [2] and is considered a major risk factor for the development of hepatocellular carcinoma [3]. Although oxidative stress [4], pro-inflammatory cytokines [5, 6], and immune response [7, 8] are associated with inflammation and fibrosis in NASH, the molecular mechanisms by which fibrosis develops and progresses in these patients remain only partially understood.

In the course of our studies on differential gene expression in NASH patients with severe fibrosis, we observed upregulation of adipocyte enhancer binding protein 1 (*AEBP1*), also known as aortic carboxypeptidase (ACLP) compared to NASH patients with no histological evidence of fibrosis [9]. These findings were consistent with those showing increased hepatic ACLP/AEBP1 protein expression with NAFLD progression in humans and mice [10]. AEBP1 is a multifunctional protein implicated in a wide range of biological processes including adipogenesis [11–13], cell differentiation [14–16], and macrophage cholesterol homeostasis [17], although a role for this protein in forming collagen-rich tissues and in the development of tissue fibrosis is also emerging. Mutations in the *AEBP1* gene can cause an inherited connective tissue disorder [18]. In patients with idiopathic pulmonary fibrosis and mice with bleomycin-induced pulmonary fibrosis, *AEBP1* expression is upregulated in lung tissue [19], where it controls myofibroblast differentiation through a mechanism involving activation of transforming growth factor beta receptors, stimulation of SMAD3, enhanced expression of smooth muscle actin, and subsequent collagen production [19]. *AEBP1* was recently found to be expressed in hepatic stellate cells (HSCs) and shown to complex with frizzled-8 and low-density lipoprotein-related receptor 6 to activate canonical WNT signaling, resulting in activation of HSCs in mouse models of NASH [10]. In parallel, *AEBP1* was identified as a key control gene of a NASH co-regulatory network constructed using publicly available microarray data [20]. The same group also showed that in the presence of a high fat/high cholesterol diet, ApoE^-/-^ mice with NASH showed higher hepatic *AEBP1* expression relative to animals with NAFLD [20]. These aggregate findings suggest a role for *AEBP1* in the development of fibrosis within the pathological context of NASH.

Here we sought to further investigate the relationship between *AEBP1* and hepatic fibrosis in NASH. *AEBP1* expression paralleled worsening severity of fibrosis in NASH patients, was upregulated in human hepatic stellate cell activation, and modulated by glucose, fructose, and palmitate. We found that *AEBP1* regulates the expression of genes involved in extracellular matrix (ECM) maintenance, as well as a number of predicted target genes that were differentially expressed in NASH fibrosis. We identified and confirmed functional interactions between miR-372-3p and miR-373-3p and the *AEBP1* 3’-untranslated region and demonstrated that both miRNAs regulate AEBP1 expression in liver cells. These results implicate *AEBP1* in a novel pathway associated with the development of hepatic fibrosis in NASH.

## Materials and methods

### RNA sequencing data analysis

Details of patient samples, RNA sequencing experiments, and raw data processing are provided elsewhere [9]. Previously generated RNA-sequencing data are freely available in the NCBI Bioproject database (https://www.ncbi.nlm.nih.gov/bioproject/512027). In our analyses here, we filtered the raw count table data to include only genes with an average count greater than five across all samples. We normalized raw counts using the *voom* algorithm [21] and adjusted for batch effects using the *ComBat* algorithm [22]. Following removal of outliers, the final sample size across the four histological grades was normal (n = 36), steatosis (n = 50), inflammation (n = 52), and fibrosis (n = 53). We performed a pairwise comparison of *AEBP1* expression in severe fibrosis versus the other histological classes (i.e., normal, steatosis, or inflammation) using bivariate logistic regression. To investigate the relationship between *AEBP1* gene expression and histological grade of fibrosis, we modeled an ordinal logistic regression considering grade as a dependent variable, adjusting for sex and age. P values were adjusted for multiple comparisons using the Bonferroni method (Padj). All analyses were conducted using R.

### RNA extraction from liver wedge biopsies

Liver wedge biopsies were obtained from individuals enrolled in the Bariatric Surgery Program at the Geisinger Clinic Center for Nutrition and Weight Management [23]. Details of the study population can be found elsewhere [24–26] and in S1 Table. All study participants provided written informed consent for research, which was conducted according to The Code of Ethics of the World Medical Association (Declaration of Helsinki). The Institutional Review Boards of Geisinger Health System, Translational Genomics Research Institute, and Temple University School of Medicine approved the research protocol. Approximately 5–10 mg of biopsied liver tissue was homogenized in lysis buffer (Qiagen; Germantown, MD). Total RNA was extracted from homogenized lysate using the RNeasy Mini Kit (Qiagen) and quantified using the NanoDrop One spectrophotometer (Applied Biosystems; Foster City, CA).

### Cell culture

LX-2 cells (Merck Millipore; Billerica, MA) were cultured in Dulbecco’s Modified Eagle Medium (DMEM) (Thermo Fisher Scientific; Waltham, MA) supplemented with 2% fetal bovine serum (FBS) and 1% Pen/Strep (Omega Scientific; Tarzana, CA). Approximately 1 x 10^6^ cells were thawed in T-75 flasks (Corning Life Sciences; Corning, NY) containing 12 mL cell culture medium and placed at 37°C in a Hera Cell 5% CO_2_ incubator (Thermo Fisher Scientific). Culture medium was replaced the first day after thawing, and then every 72 hours until 80% confluent. HepG2 and HEK293 cells (ATCC; Manassas, VA) were cultured in Dulbecco’s Modified Eagle Medium (DMEM) (Thermo Fisher Scientific) supplemented with 10% fetal bovine serum (FBS) and 1% Pen/Strep (Omega Scientific). Culture medium was replaced the first day after thawing, and then every 48 hours until 80% confluent. Primary human hepatocytes (Thermo Fisher Scientific) were thawed in 50 mL Cryopreserved Hepatocyte Recovery Medium (CHRM) and plated in 500 υL William's E Medium supplemented with Hepatocyte Plating Supplement Pack on collagen-coated 24-well plates (Thermo Fisher Scientific). Culture medium was replaced the first day after thawing with William's E Medium supplemented with Hepatocyte Maintenance Supplement Pack.

### Cell treatments

LX-2 cells were seeded at 0.5 x 10^6^ cell/well on 6-well culture dishes (VWR International; Radnor, PA) and serum-starved overnight. Cell culture medium was aspirated and replaced with DMEM, 10% FBS, 0.5 mM isobutylmethylxanthine, 1 μM dexamethasone, and 167 nM insulin [i.e., MDI solution (Sigma-Aldrich; St. Louis, MO)] for 72 hours to induce a state resembling biological quiescence [27, 28].

Primary human hepatocytes were serum-starved overnight, and then treated with 1 mM palmitate (Sigma-Aldrich) conjugated with bovine serum albumin (BSA; [Omega Scientific]), 20 mM fructose, or a combination of the two for 48 hours. Treatment with 1% BSA was included as a negative control. Oil Red O staining was used to assess lipid uptake.

### Total RNA extraction and quantification from cells

Total RNA was extracted from cells using the RNeasy mini kit (Qiagen) according to the manufacturer’s protocol. RNA quality and concentration were determined by absorbance at 260 nm using the NanoDrop One spectrophotometer (Thermo Fisher Scientific). MicroRNA was extracted from cells using the miRNeasy mini kit (Qiagen) following the product protocol. The miRNA quantity and quality were assessed using the 2100 Bioanalyzer System (Agilent Technologies; Santa Cruz, CA).

### Quantitative real-time PCR (qPCR)

The TaqMan RNA-to-Ct 1-Step kit (Thermo Fisher Scientific) in conjunction with the QuantStudio 6 Flex Real-Time PCR system (Thermo Fisher Scientific) and TaqMan commercial primers were used to measure transcript levels. MiRNA was processed using the Taqman MicroRNA Reverse Transcription Kit (Applied Biosystems), followed by amplification using commercial probes (Applied Biosystems). Cycle threshold (Ct) levels were generated using QuantStudio Real-Time PCR Software 1.0. Messenger RNA and miRNA data were normalized using glyceraldehyde 3-phosphate dehydrogenase (*GAPDH*) and *RNU6B* (both of which showed invariant expression levels), respectively. The– ΔΔCt method was used to determine fold-change of gene expression.

### Protein extraction and quantification

Cells were lysed in 350 uL RIPA buffer and lysate was transferred to 1.5 ml tubes and centrifuged at 5000 x *g* for 5 minutes at 4°C. Total protein concentrations for each sample were determined using the Pierce BCA Protein Assay kit (ThermoFisher Scientific). Levels of AEBP1 and GAPDH were detected using western blot analysis. An equal amount of protein was loaded on a NuPage pre-cast Bis-Tris protein gel with a 4–12% polyacrylamide gradient (Life Technologies), and then transferred from gels to nitrocellulose membranes (Life Technologies). Membranes were placed in Pierce Protein-Free (PBS) Blocking Buffer (ThermoFisher Scientific) for ninety minutes at room temperature, and then incubated overnight at 4°C with primary antibodies directed against ACLP/AEBP1 (anti-mouse 1:1000 dilution; Santa Cruz; Dallas, TX; catalog number: sc-271374) or GAPDH (anti-rabbit 1:1000; Cell Signaling Technology; Danvers, MA). Membranes were washed for eight minutes in 1X TBS-Tween buffer, and then incubated with secondary antibodies, either anti-mouse or anti-rabbit, labeled with horseradish peroxidase (1:3000; Cell Signaling Technology) for one hour at room temperature. Membranes were incubated with Clarity Western ECL Blotting Substrate (Bio-Rad; Hercules, CA) and protein bands were imaged using the Odyssey FC imaging system (LI-COR Biotechnology; Lincoln, NE).

### Transfection with AEBP1 siRNA

Approximately 2x10^5^ LX-2 cells/well were seeded in a 24-well plate with 6 uL Lipofectamine RNAiMAX transfection reagent (Thermo Fisher Scientific) containing either 50 nM or 100 nM Silencer Select Pre-Designed siRNA directed against *AEBP1* or Silencer Select Negative Control Number 1 siRNA (both Thermo Fisher Scientific) and incubated at 37°C. Cells were harvested after 48 or 72 hours. Total RNA was extracted and expression levels of the genes of interest were assessed as described.

### Prediction of miRNAs targeting the 3’ untranslated region (3’UTR) of AEBP1

Potential *AEBP1*-targeting miRNAs were identified using four prediction algorithms (miRWalk, miRanda, RNA22, and Targetscan) as implemented in miRWalk 2.0 (http://zmf.umm.uni-heidelberg.de/apps/zmf/mirwalk2/). The miRmap tool [29] was used to predict miRNA target repression strength. MiRNA conservation was determined using the phyloP (“phylogenetic *P*-values”) program (http://compgen.bscb.cornell.edu/phast).

### 3’UTR dual-luciferase reporter assay

The 3’ untranslated region (3’UTR) of *AEBP1* and a scrambled sequence insert were generated by PCR and cloned into the multiple cloning site (MCS) of the pmirGLO vector (Promega Corp; Madison, WI) by the Emory Integrated Genomics Core (Atlanta, GA). The resulting (pGLO-AEBP1) and (pGLO-Scramble) reporter plasmids were sequence-verified. Approximately 2x10^5^ HEK293 cells/well were seeded in a 24-well plate, co-transfected with 2 μg of the indicated vector and 10 pM miR-372-3p mimic, miR-373-3p mimic, or negative control mimic using 4 ul Lipofectamine 2000 per well (Thermo Fisher Scientific), and then incubated at 37°C for 48 hours. Luciferase activity was measured using the Dual-Glo Luciferase Assay System (Promega). Luminescence was detected using the EnVision 2105 Multimode Plate Reader (PerkinElmer; Waltham, MA). The ratio of firefly luciferase activity to Renilla luciferase activity was used to calculate normalized luciferase activity. The effect of miR-372-3p and miR-373-3p on gene expression was determined by setting the normalized luciferase activity of the negative mimic control + pGLO-AEBP1 3’UTR vector to 100% and showing the normalized luciferase activity of miR-372-3p mimic or miR-373-3p mimic + AEBP1 3’UTR vector as a percentage. For the control conditions, relative luciferase activity was determined by showing the normalized luciferase activity of miR-372-3p mimic or miR-373-3p mimic + pGLO-Scramble vector as a percentage of the negative mimic control + vector control. The Mann-Whitney U test was used to assess differences between conditions.

### Transfection with miRNA mimics and inhibitors

Approximately 2x10^5^ LX-2 cells/well were seeded in a 24-well plate with 6 uL Lipofectamine RNAiMAX transfection reagent (Thermo Fisher Scientific) containing either 75 nM *mirVana miR-372-3p*/*miR-373-3p* mimic, 75 nM anti-miR-372-3p/miR-373-3p inhibitor, or 75 nM scrambled sequence control (*mir*Vana Negative Control #1 [Thermo Fisher Scientific]) and incubated for 48 hours at 37°C. Total RNA was extracted using the miRNeasy kit (Qiagen; Germantown, MD) and miRNA over-expression and knockdown was verified using qPCR.

### Statistical analysis

All statistical analyses were performed using GraphPad Prism 8 (GraphPad Software; La Jolla, CA). Data were analyzed as the mean ± standard deviation (SD) from at least three independent assays. The Kruskal-Wallis one-way analysis of variance test with a Dunn post-hoc test was used to assess differences between conditions, unless otherwise indicated. A *P*-value <0.05 was considered statistically significant.

## Results

### AEBP1 expression is upregulated in NASH fibrosis

**Fig 1:**
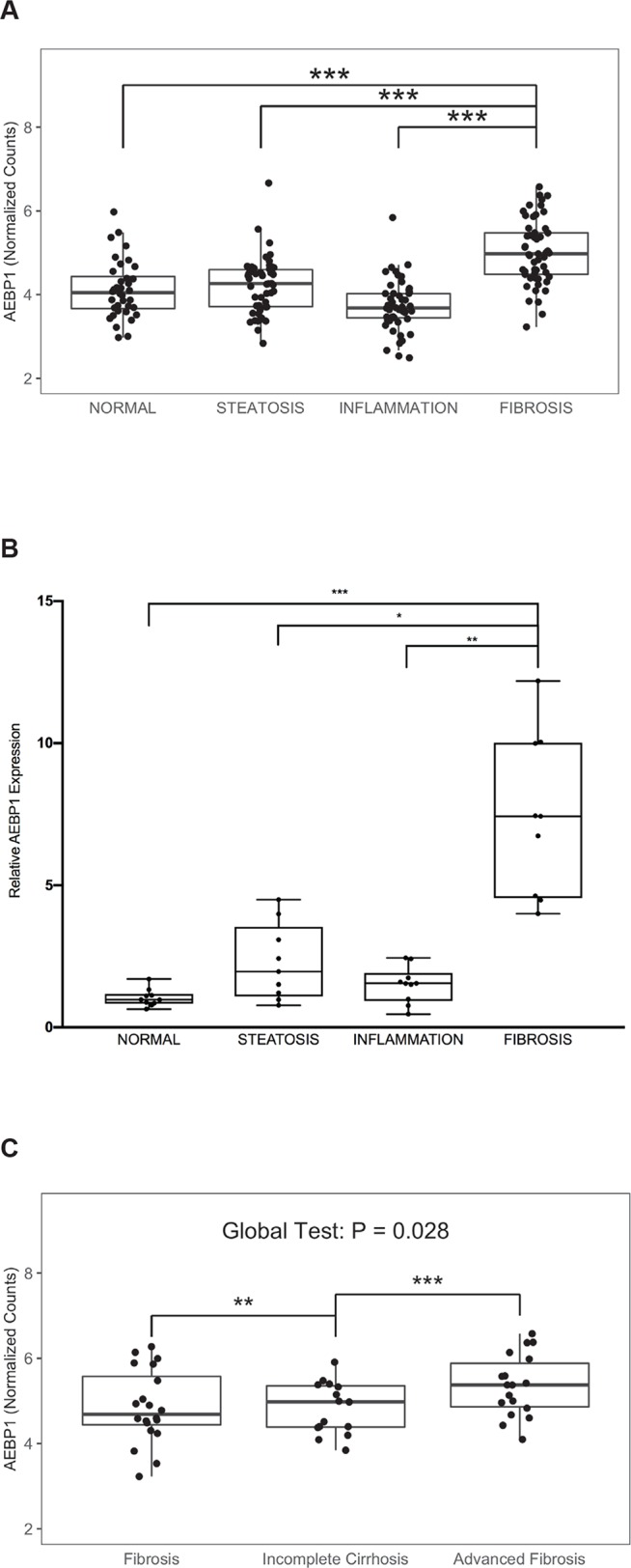
Hepatic *AEBP1* expression in NAFLD patients. **A)** Using RNA-sequencing data [9], we determined average *AEBP1* transcript abundance (normalized counts) in NAFLD samples across a spectrum of liver histology (36 normal, 50 steatosis, 52 inflammation, and 53 fibrosis). The ordinal logistic regression showed a significant positive trend (β = 0.244; P = 4.8E-4), whereas the pairwise comparisons conducted with logistic regression showed a significant difference between normal and fibrosis histological grades (Padj = 1.3E-03). **B)** *AEBP1* gene expression was measured in a subset of NAFLD patients with steatosis (n = 10), lobular inflammation (n = 10), cirrhosis (n = 10), and normal liver histology (n = 10). Real time qPCR was performed using Taqman gene expression assays for *AEBP1* and *GAPDH*. The Kruskal-Wallis one-way analysis of variance test with a Dunn post-hoc test was used to assess differences in expression between fibrosis and the other histological grades. *P≤0.05, **P≤0.001, and *** P≤ 0.0001. **C)** Using RNA-sequencing data (9), we determined *AEBP1* transcript abundance (normalized counts) in human NAFLD samples with bridging fibrosis (n = 20), incomplete cirrhosis (n = 15), and advanced fibrosis (n = 18). The ordinal logistic regression adjusted for sex and age showed a global significant positive trend (beta = 0.906; P = 0.028), with the median expression level across different stages of fibrosis.

We previously reported upregulated hepatic *AEBP1* expression in NASH patients with fibrosis compared to those with NASH but no histological evidence for fibrosis identified using massively parallel RNA-sequencing [9]. These results are concordant with those of Teratani et al [10], who recently reported enhanced AEBP1 expression in liver tissue from NASH patients (N = 44) compared to individuals with NAFLD (N = 16) and metastatic liver cancer patients with no evidence of steatosis, inflammation, or fibrosis (N = 14). Here we assessed *AEBP1* expression in NASH fibrosis versus other NAFLD histological classes (S1 Table) using pairwise comparisons. We observed significant differences in hepatic *AEBP1* expression between fibrosis and normal (Padj = 1.5E-05), steatosis (Padj <0.001), and inflammation (Padj <0.001) (Fig 1A). Analysis of *AEBP1* expression using quantitative RT-PCR in a subset of NAFLD patients validated genome-wide results (Fig 1B). We then examined the relationship across fibrosis stage using an ordinal logistic regression (adjusting for sex and age) in an analysis of the RNA-sequencing data. We observed a significant trend of increasing *AEBP1* levels with increasing severity of fibrosis with advanced fibrosis (i.e., cirrhosis) > incomplete cirrhosis > bridging fibrosis (beta = 0.906; P = 0.028). The median expression level increased from 4.69 (bridging fibrosis) to 4.97 (incomplete cirrhosis) to 5.37 (cirrhosis). **(**Fig 1C). The partial regression P values for bridging fibrosis vs incomplete fibrosis, and incomplete cirrhosis vs cirrhosis were statistically significant (P < 0.01).

### AEBP1 expression is upregulated in activated hepatic stellate cells

**Fig 2:**
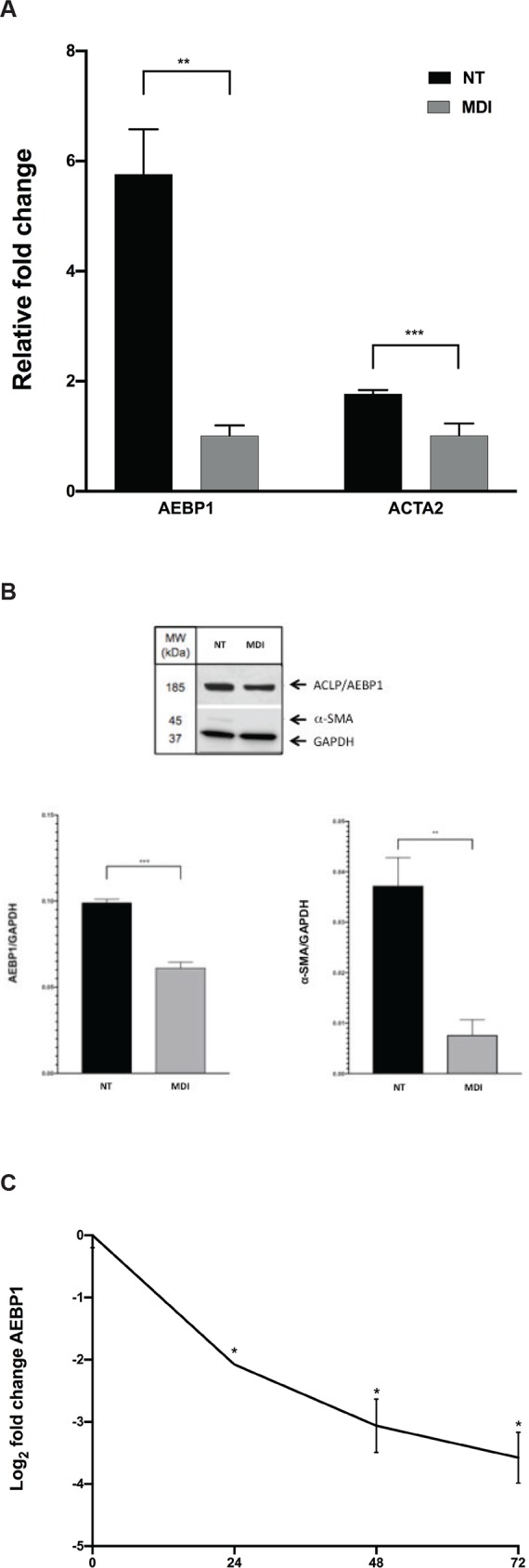
AEBP1 expression in untreated and MDI-treated LX-2 cells. **A)** *AEBP1* and *ACTA2* mRNA levels were measured by qPCR in no treatment controls (NT; i.e., activated) and MDI-treated (i.e., quiescent) LX-2 cells. *AEBP1* and *ACTA2* levels were normalized to *GAPDH* for RNA and protein expression analysis. **B)** Protein levels of AEBP1, αSMA, and GAPDH were assayed using western blot. Imaging was performed and used for densitometry. **C)** Time course of *AEBP1* expression over 72 hours of MDI treatment. Transcript levels of *AEBP1* expression were normalized to *GAPDH*. All experiments were performed in triplicate. *P≤0.05.

Findings of increased hepatic *AEBP1* expression in NASH patients with fibrosis led us to ask whether the gene was differentially regulated during myofibroblastic transformation of hepatic stellate cells (HSCs), the key fibrogenic cells of the liver. We compared *AEBP1* expression levels in HSCs (LX-2 cells) grown on plastic to achieve a myofibroblast phenotype or treated with MDI solution to induce a state resembling biological quiescence [30]. Increased *ACTA2*/αSMA expression was indicative of LX-2 cell activation (Fig 2A and 2B). In untreated LX-2 cells, AEBP1 transcript and protein expression was 5.8-fold and 44% higher, respectively, compared to those treated with MDI solution (Figs 2A and 2B and S1). The form of the ACLP/AEBP1 protein (~185 kDA) detected was the same as that reported earlier [10]. We also observed that *AEBP1* expression decreased over time in response to MDI treatment, showing a 2.1-, 3.1-, and 3.6-fold (log_2_) reduction at 24, 48, and 72 hours, respectively (Fig 2C). In LX-2 cells grown on Matrigel-coated plates, *AEBP1* levels decreased in a manner similar to that seen in MDI-treated cells (S2 Fig).

### Effect of glucose, palmitate, and fructose on AEBP1 expression in liver cells

**Fig 3:**
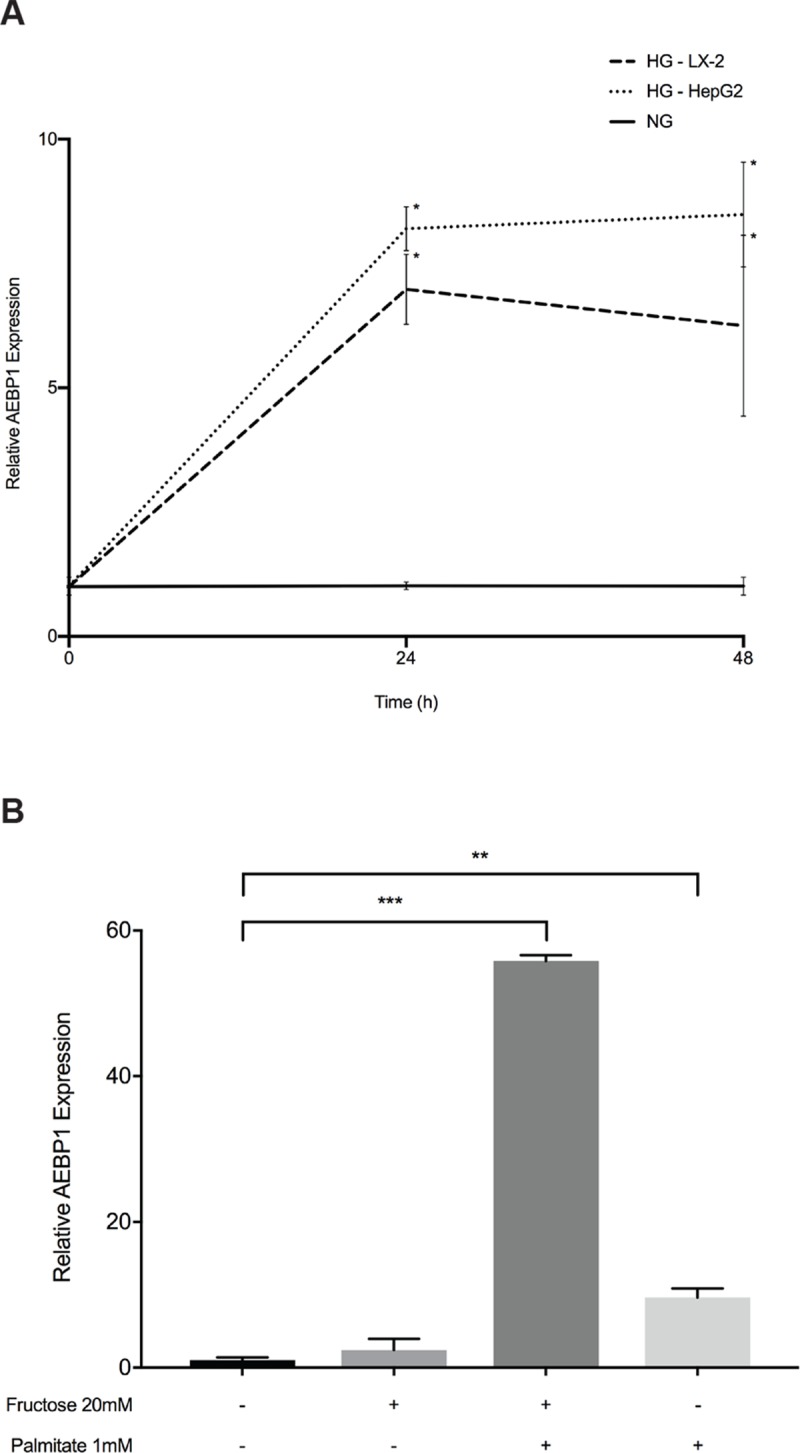
Effect of glucose, palmitate, and fructose on *AEBP1* expression. LX-2 and HepG2 cells were serum-starved for 24 hours to arrest and synchronize cell growth. After this time, cells were grown in medium supplemented with 5% FBS, containing either normal glucose (NG: 5.5 mM) or high glucose (HG: 25 mM) for 24 or 48 hours. **A)** Relative quantification of *AEBP1* transcript levels was performed using quantitative PCR. *AEBP1* expression levels were normalized using *GADPH*, setting values to one under NG conditions at each time point. Data are presented as mRNA fold-increase incubated under HG or NG conditions. Results represent average of three independent experiments. Data are mean ± SD.*P<0.05. **B)** Primary human hepatocytes were treated with 1 mM BSA-conjugated palmitate, 20 mM fructose, or a combination of the two for 48 hours. The black bar represents treatment with BSA-only. All experiments were performed in triplicate. Data are mean ± SD. Differences between conditions were assessed using one-way ANOVA test with a Dunnett’s multiple comparison post-test. **P<0.005; ***P<0.0001.

Because of the close association between type 2 diabetes and NAFLD, we investigated the effects of glucose in LX-2 cells and HepG2 cells, both of which are widely used for in vitro studies of NASH. In both cell types, *AEBP1* expression increased >7.0—fold over 24 hours and remained upregulated up to 48 hours under high glucose (HG: 25 mM) compared to normal glucose (NG: 5 mM) conditions (Fig 3A). We have previously observed effects of lipid loading on target gene expression in activated HSCs [31] and in primary cultured murine HSCs, palmitate was observed to additively increase *AEBP1* expression [10]. To extend these studies to hepatocytes, we measured the effect of lipid loading on *AEBP1* expression in primary human hepatocytes treated with 1 mM palmitate. In these cells, *AEBP1* expression increased 9.6-fold compared to vehicle (Fig 3B). We next sought to investigate the effects of fructose on *AEBP1* expression given the relationship between high fructose consumption and severity of NAFLD [32] and found that *AEBP1* expression increased 2.4-fold compared to the control cells (Fig 3B). Interestingly, in response to treatment with a mixture of 1 mM palmitate and 20 mM fructose, we saw a large, non-additive effect (>55.8-fold increase) on *AEBP1* expression.

### AEBP1 regulates predicted target genes uniquely associated with NASH fibrosis

**Fig 4:**
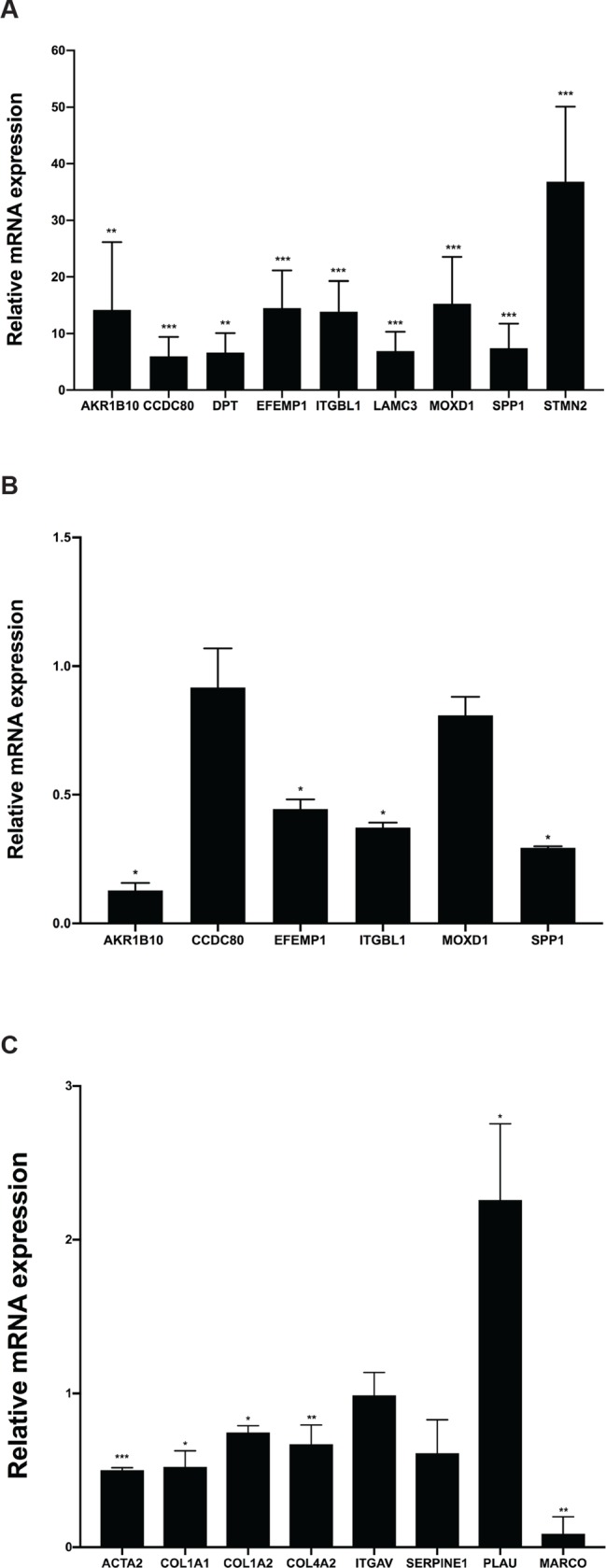
Expression of predicted AEBP1 target genes. **A**) We measured levels of the genes of interest using qPCR and hepatic RNA from NAFLD patients with normal liver histology and severe fibrosis. Transcript levels were normalized to *GAPDH*, which showed invariant expression. Effects of *AEBP1* knockdown on **B)** fibrosis-specific predicted target genes and **C)** predicted target genes involved with ECM maintenance. Data are presented as relative fold-change. A Mann-Whitney U test was performed to assess statistical significance. *P≤0.05, **P≤0.001, and *** P≤ 0.0001.

In a recent study, we identified 34 transcripts that were differentially expressed in NASH patients with or without fibrosis [9], nine of which overlapped with predicted AEBP1 target genes identified by bioinformatic analysis [20]. We first confirmed increased expression of these genes in an independent sample of NASH patients with fibrosis relative to normal histology (Fig 4A). To establish an interaction between AEBP1 and these predicted targets, we knocked down *AEBP1* expression and measured levels of the nine genes in LX-2 cells. Knockdown efficiency of AEBP1 was verified through qPCR analysis (S3 Fig). As shown in Fig 4B, in the presence of *AEBP1* siRNA, we observed a reduction of aldo-keto reductase family 1 member 10 (*AKR1B10*; 7.8-fold), EGF containing fibulin extracellular matrix protein 1 (*EFEMP1*; 2.3-fold), integrin beta-like protein 1 (I*TGBL1*; 2.7-fold), and secreted phosphoprotein 1 (*SPP1*; 3.4-fold). Expression levels of dermatopontin (*DPT*), laminin subunit gamma-3 (*LAMC3*), and stathmin 2 (*STMN2*) were not detectable in LX-2 cells.

To determine the role of *AEBP1* in the accumulation of ECM proteins, we reduced *AEBP1* expression using siRNA and quantified levels of selected genes involved in ECM structure and maintenance by qPCR. We also analyzed levels of integrin subunit alpha V (*ITGAV*), collagen type IV alpha 2 chain (*COL4A2*), and macrophage receptor with collagenous structure (*MARCO*), which encode known cell adhesion proteins and are predicted AEBP1 target genes [20], although these genes were not part of the set of fibrosis-specific genes identified in our previous work [9]. As shown in Fig 4C, levels of *COL4A2* and *MARCO* were decreased 1.5-fold and 11.3-fold, respectively, compared to levels obtained in cells transfected with the negative control siRNA. Given the upregulation of *AEBP1* observed in activated HSCs, we also investigated effects of *AEBP1* depletion on expression of selected genes known to be affected by myofibroblastic transition [30]. We found that knockdown of *AEBP1* expression resulted in 2.0-fold, 1.9-fold, 1.3-fold, and 1.6-fold (log_2_) decrease in levels of actin alpha 2 smooth muscle (*ACTA2*), collagen type 1 alpha 1 chain (*COL1A1*), collagen type 1 alpha 2 chain (*COL1A2*), and serpin family E member 1 (*SERPINE1*) expression, respectively, and a 2.3-fold increase in urokinase plasminogen activator (PLAU) expression (Fig 4C).

### AEBP1 is regulated by miRNAs downregulated in NASH fibrosis

**Fig 5:**
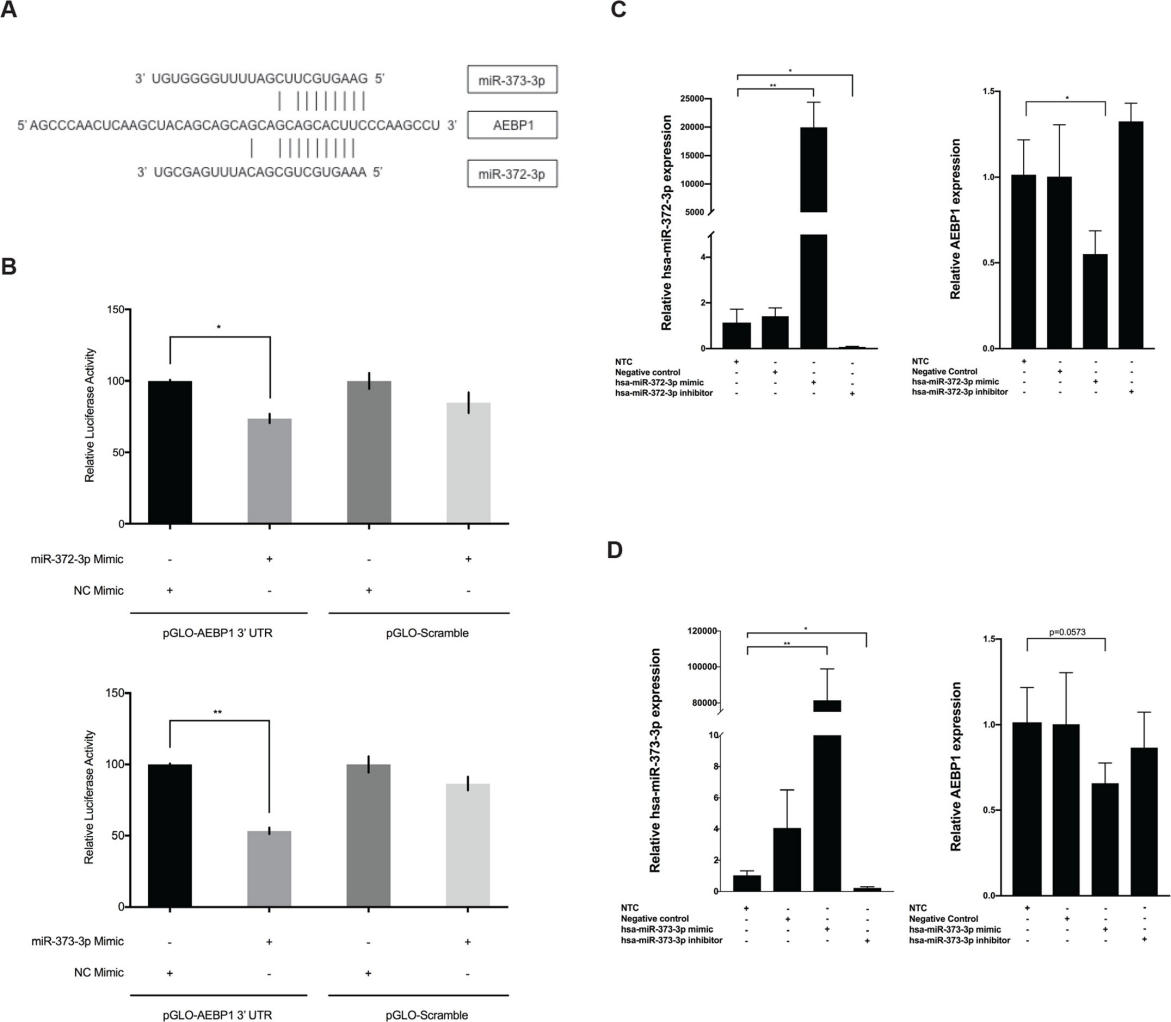
miR-372-3p and miR-373-3p functionally interact with *AEBP1* to regulate its expression. **A)** The target site for miR-372-3p and miR-373-3p was predicted to align to positions 3829–3835 in the *AEBP1* 3’UTR (GenBank Accession NM_001129.4). Vertical lines indicate paired alignment between the miRNA seed region and *AEBP1* 3’UTR sequence. **B)** Approximately 20,000 human embryonic kidney 293 (HEK293) cells were co-transfected with the pGLO-AEBP1 3’UTR vector or scrambled control vector (pGLO-Scramble) and miR-372-3p mimic, miR-373-3p mimic, or scrambled sequence control miRNA (negative control [NC] mimic), and luciferase activity was measured 48 hours post-transfection using the Dual-Glo Luciferase Assay System (Promega). The ratio of firefly luciferase activity to renilla luciferase activity was determined for each sample and the firefly/renilla relative luminometer units (RLU) in HEK293 cells co-transfected with miRNA mimic and the pGLO-AEBP1 3’UTR vector were compared with those from cells co-transfected with pGLO-Scramble and NC mimic. The Mann-Whitney U test was used to assess differences between conditions; differences between the negative controls were not statistically significant. For functional studies, activated LX-2 cells were transfected with 75 nM *mirVana miRNA-372-3p/miR-373-3p* mimic, 75 nM anti-miR-372-3p/miR-373-3p inhibitor, or 75 nM scrambled sequence control. After 48 hours cells were harvested and total RNA was extracted. The TaqMan RNA-to-Ct 1-Step kit (Thermo Fisher Scientific) and TaqMan commercial primers were used to measure **C)** miR-372-3p and *AEBP1* levels and **D)** miR-373-3p and *AEBP1* levels in the presence of the respective mimic and siRNA. Cycle threshold levels were generated using QuantStudio Real-Time PCR Software 1.0. miRNA and mRNA data were normalized using *RNU6B* + *RNU48* and *GAPDH*, respectively. Black bars represent the scrambled miRNA negative control, while the gray bars represent treatment conditions. All experiments were performed in triplicate. Data are means ±SD. A paired t-test was used to compare the treatment group to the no treatment control group (NTC) *P<0.05; **P<0.001; ***P<0.0001.

In colorectal cancer cells, *AEBP1* expression is negatively regulated by miR-214 [33]. To explore potential mechanisms of miRNA-mediated regulation of *AEBP1* in NASH fibrosis, we analyzed the *AEBP1* 3’UTR to identify putative miRNA binding sites using a comparative platform comprised of four different algorithms (see Methods section). Seven miRNA binding site predictions were identified using all four algorithms (S2 Table). Two of these, miR-372-3p and miR-373-3p were found to be downregulated in NAFLD patients with fibrosis compared to those with normal histology: miR-372-3p (log_2_ fold-change = -1.7; Padj = 0.031) and miR-373-3p (log_2_ fold-change = -2.4; Padj = 0.001) [24]. Levels of miR-372-3p were 2.1-fold higher, while those of miR-373-3p were 2.9-fold lower, in quiescent versus activated LX-2 cells, respectively (S4 Fig). According to miRmap [29], miR-372-3p and miR-373-3p share overlapping binding sites at positions 3829–3835 bp (NM_001129) in the *AEBP1* 3’UTR (Fig 5A). To validate this interaction, we used a dual luciferase reporter assay to measure direct binding between the two miRNAs and the *AEBP1* 3’UTR. Co-transfection of a construct containing the *AEBP1* 3’UTR and exogenous miR-372-3p or miR-373-3p resulted in a 26% and 46.7% reduction in relative luciferase activity compared to control conditions, respectively (Fig 5B).

We next transfected LX-2 cells with mimics or siRNAs to determine possible functional consequences of miR-372-3p and miR-373-3p on *AEBP1* expression. As expected, transfection with miRNA mimic or inhibitor corresponded with an increase or reduction, respectively, in miR-372-3p (Fig 5C) and miR-373-3p (Fig 5D). In the presence of miR-372-3p mimic, *AEBP1* expression was reduced 1.8-fold (p<0.05). miR-372-3p inhibitor increased *AEBP1* expression, but the change was not statistically significant (Fig 5C). Treatment of LX-2 cells with miR-373-3p mimic reduced *AEBP1* levels 1.5-fold (P = 0.0573), although the presence of miR-373-3p inhibitor did not significantly alter *AEBP1* expression.

## Discussion

Given the strong link between fibrosis and risk of liver-related mortality in NAFLD patients [34, 35], efforts to identify and characterize the specific mechanisms contributing to NAFLD progression are critical for the development of effective therapeutic and preventive strategies. We previously identified 34 genes that were upregulated in NASH fibrosis, relative to non-fibrotic NASH. One of these genes, *AEBP1*, was recently reported to be a potential central regulator driving the transition of NASH, possibly through the modulation of target genes [20]. One of the major findings of the current work demonstrated that *AEBP1* regulates the expression of nine algorithm-predicted target genes that overlapped with our set of fibrosis-specific genes, including *AKR1B10*, *CCDC80*, *DPT*, *EFEMP1*, *ITGBL1*, *LAMC3*, *MOXD1*, *SPP1*, and *STMN2*. We also showed that *AEBP1* regulates expression of genes known to play a role in ECM production and maintenance, concordant with recent findings from a comprehensive analysis of AEBP1 in mouse models of NAFLD [10]

We found that hepatic *AEBP1* levels were elevated in fibrotic tissue compared to other non-fibrotic histologic grades. We also observed a significant trend of increasing AEBP1 expression concomitant with worsening severity of fibrosis with the highest hepatic levels found in patients with advanced fibrosis. Teratani et al [10] found increased hepatic AEBP1 staining in NASH patients compared to NAFLD patients. In that study, however, AEBP1 expression relative to fibrosis was not investigated; thus, the current results extend these findings to indicate that *AEBP1* expression in the liver parallels the onset of fibrosis in NASH and suggest that *AEBP1* may represent a specific therapeutic target to prevent the development of NASH fibrosis.

The presence of increasing AEBP1 levels in parallel with worsening fibrosis is consistent with changes in *AEBP1* expression occurring with transdifferentiation of LX-2 cells, an immortalized human hepatic stellate cell line that retains important features of primary hepatic stellate cells [30]. We observed that *AEBP1* levels decreased in a time-dependent manner in LX-2 cells treated with MDI solution or grown on Matrigel to induce quiescence compared to cells grown on plastic that develop a myofibroblastic phenotype. These findings are in contrast to those showing more abundant AEBP1 expression in quiescent rat aortic smooth muscle cells compared to actively proliferating cells [15]. However, a recent study demonstrated that recombinant AEBP1 enhanced myofibroblast differentiation and promoted fibrogenesis [14], consistent with our results in LX-2 cells. Further, AEBP1 expression was elevated in HSCs concomitant with NAFLD progression [10], indicating that increased, not decreased, expression occurs with myofibroblastic activation. Importantly, in that work HSC-specific AEBP1-knockout mice developed NASH following a high fat/high cholesterol diet for 24 weeks, but exhibited significantly less fibrosis compared to AEBP1-floxed animals under the same conditions. Repression of AEBP1 expression also inhibited HSC activation and significantly decreased expression of genes involved with ECM [10]. Together, these findings support a role for AEBP1 in HSC activation and initiation of fibrosis, a process that may involve increased STAT3 signaling in response to elevated free fatty acids, leptin, and IL-6 [10].

Previous work reported that AEBP1 expression was restricted to stellate cells and not observed in hepatocytes [10] using immunohistochemistry to distinguish cell specificity. In the current work, we detected *AEBP1* expression in primary human hepatocytes and in the HepG2 hepatoma cell line. These discrepant findings may be attributed to differences in measurement: immunohistochemistry to visualize protein expression is less sensitive than PCR or sequencing to detect mRNA. In addition, AEBP1 is a secreted protein, thus intracellular levels may be low relative to its location in the extracellular matrix.

To extend *AEBP1* expression findings from human liver biopsy tissue, we used *in vitro* models of diabetes and NAFLD by assaying the effects of glucose, fructose, and palmitate treatments on human stellate and liver cells. There is a strong association between diabetes and liver fibrosis, with a much higher risk of progression of NAFLD in patients with diabetes [36]; thus, glucose metabolism appears to play a role in the development of steatohepatitis and fibrosis. Like glucose, fructose has been implicated in NAFLD. Daily consumption of fructose was associated with a higher stage of fibrosis and a lower grade of steatosis in patients with NASH [32]. In mice, fructose, combined with a diet high in fat and cholesterol, can induce NASH and hepatic fibrosis within four to six months [37]. Our findings that glucose, fructose, and palmitate upregulate *AEBP1* expression are consistent with a role for *AEBP1* in the underlying mechanism (s) of these risk factors. However, the mechanism (s) by which these factors upregulate *AEBP1* expression is not yet known. In primary human hepatocytes, palmitate and palmitate plus high glucose were both found to induce expression of the fatty acid binding protein (*FABP4*) gene [38]. AEBP1 binds to the proximal promoter of FABP4 [39], suggesting it may have a role in the response to palmitate and glucose. AEBP1 also interacts with tumor suppressor phosphatase and tensin homolog deleted on chromosome ten (PTEN), which is a negative regulator of insulin signaling and insulin sensitivity in adipose tissue [40]. Saturated fatty acids increase PTEN levels [41], which may be another pathway by which palmitate influences *AEBP1* expression. Why fructose and palmitate together caused a dramatically higher level of upregulation than either treatment alone warrants further exploration; however, it is possible that AEBP1 may be a common regulatory point for gene expression changes induced by both treatments.

Another common regulatory point may be WNT signaling. Interestingly, AEBP1 has been shown to be associated with activation of the canonical WNT pathway in HSCs [10]. Activation of this pathway causes a methylation-dependent downregulation of PPARγ, which is involved in HSC activation [42]. Increased AEBP1 expression from exogenous sugars or fats could induce canonical WNT pathway signaling that would reduce PPARγ promoter activity and subsequent expression in HSCs, leading to decreased PPARγ expression and resultant HSC activation. This is consistent with the observed synergistic effect of a high fat diet with fructose on decreasing PPARγ expression in mice [43]. However, palmitic acid was also observed to increase PPARγ expression in HepG2 cells, suggesting that stellate cells may possess a response to increased fatty acid exposure that is distinct from hepatocytes.

We used a combination of four predictive algorithms to identify miR-372-3p and miR-373-3p as potential *AEPB1*-targeting miRNAs and found that both miRNAs negatively regulated *AEBP1* expression. Both miR-372-3p and miR-373-3p were significantly downregulated in NAFLD patients with fibrosis compared to those with normal histology [24], which would be consistent with upregulated *AEBP1* expression. Other studies have reported involvement of both miRNAs in human malignant tumors through the targeted downregulation of a number of genes including fibroblast growth factor 9 [44], dickkopf WNT signaling pathway inhibitor 1 [45], and others [46–48], and miR-372-3p was shown to promote epithelial mesenchymal transition in breast carcinoma through WNT pathway activation [46]. miR-372-3p and miR-373-3p share overlapping binding sites at position 3829–3835 bp in the *AEBP1* 3’UTR. This position is proximal to a binding site for miR-214 (3842–3848 bp), which was found to directly target and downregulate *AEBP1* expression in colorectal cancer (HT-29) cells [33]. The presence of clustered functional miRNA binding sites indicates that this region may be important for the regulation of *AEBP1*. More detailed studies involving mutagenesis of the predicted sites will be needed to further characterize the post-transcriptional miRNA regulation of *AEBP1*.

**Fig 6:**
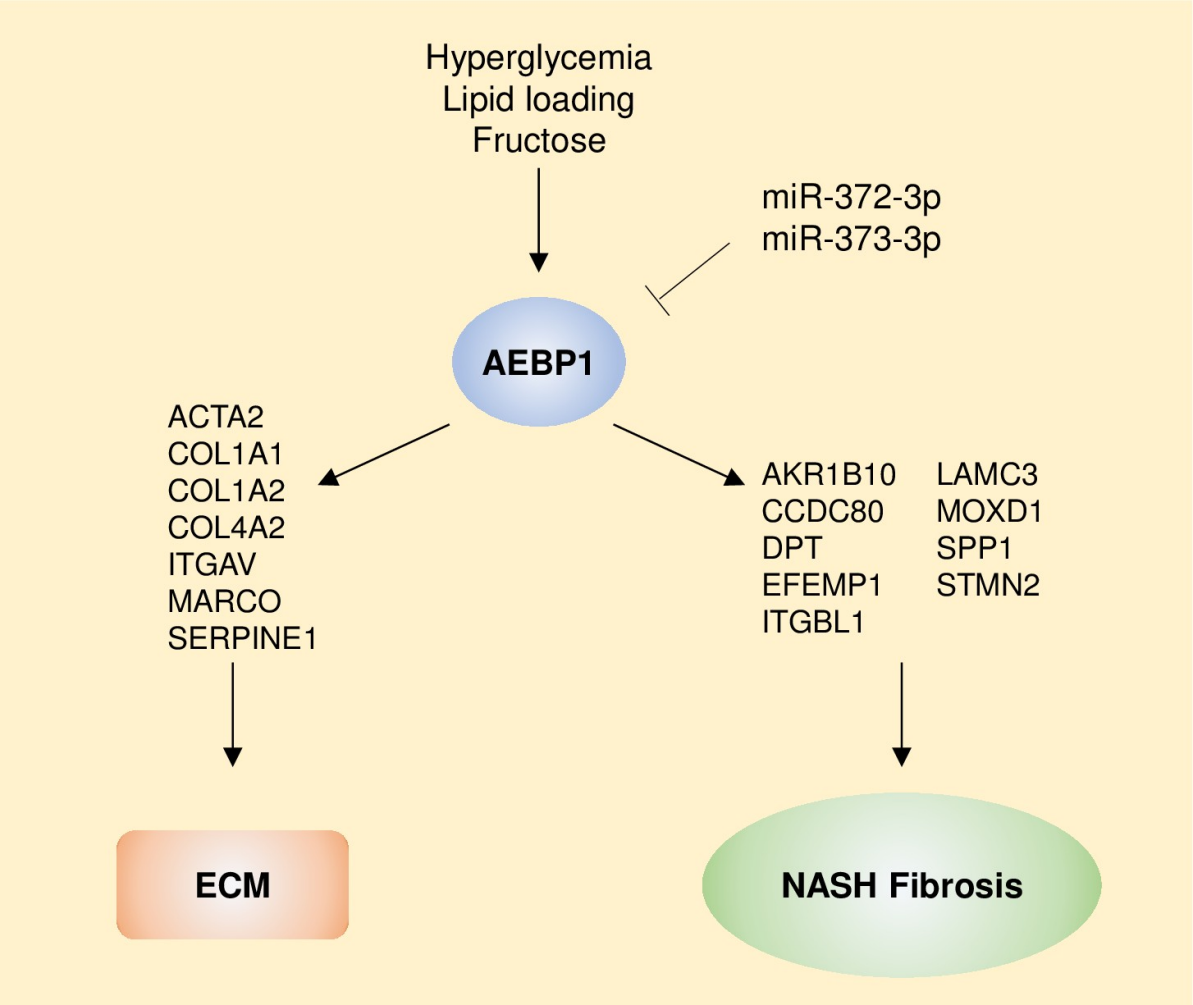
Potential mechanism by which AEBP1 affects the development of fibrosis in NASH. Hyperglycemia, lipid loading, and fructose treatment cause an increase in *AEBP1* expression in liver cells. miR-372-3p and miR-373-3p, which are reduced in NAFLD patients with advanced fibrosis, functionally interact with *AEBP1* to downregulate its expression. Increased expression of *AEBP1* is associated with altered expression of key components of the ECM and algorithmically predicted target genes belong to a core set of fibrosis-specific genes previously identified by the authors.

The current findings add to our understanding of the role of AEBP1 in hepatic fibrosis within the context of NASH in several ways. First, AEBP1-mediated regulation of fibrosis-specific genes, as well as those involved in ECM production and maintenance, suggest that this protein contributes to hepatic fibrosis through modulation of a gene network that may be specific to HSCs. Second, obesity-related factors that have also been linked to NAFLD, including glucose, fructose, and palmitate, increase AEBP1 expression, thereby exacerbating expression of these genes. Finally, miR-372-3p and miR-373-3p, which may function to downregulate AEBP1 and are known to regulate canonical WNT signaling [45, 46], are reduced in NASH patients with advanced fibrosis. Together, these results provide a potential mechanism by which AEBP1 may contribute to NASH-related fibrosis (Fig 6).

The multicellular nature of biopsied tissue limits the interpretation of differential gene expression using RNA from intact liver. We were able to demonstrate patterns of *AEBP1* expression similar to human liver in human stellate, HepG2, and primary human hepatocyte cell cultures, but we cannot exclude the possibility that other resident hepatic cell types may also contribute to its differential regulation with fibrosis. *AEBP1* is expressed by perivascular and vascular cells, as well as the stromal-vascular fraction of white adipose tissue [14]. Whether these or other cell types in the liver express *AEBP1* will require *in situ* hybridization or single cell analysis. We also chose to focus our studies on human tissue and cells. Despite the apparent phenotypic aspects of various mouse models that can be observed to parallel human NASH, there appears to be little mechanistic overlap at the level of gene expression [49]. Human studies of NAFLD and NASH are unfortunately limited by the difficulties in obtaining tissue for analyses, thus other models such as organoids [50], may be required to more faithfully recapitulate NAFLD and NASH for gene expression studies.

In summary, we found that *AEBP1* expression was increased in human liver biopsies from patients with NASH fibrosis, in activated human stellate cells, and in human liver cells treated with glucose, fructose, and palmitate. *AEBP1* regulated the expression of nine fibrosis-specific genes that were also members of an algorithm-predicted *AEBP1* target gene network in NASH. We also found that *AEBP1* functionally interacted with two miRNAs. Taken together, these findings support the idea that *AEBP1* may be a central regulator of a complex fibrosis gene expression network in human liver.

## Supporting information

S1 Fig Full western blots for ACLP/AEBP1 and α-SMA analyses shown in Fig 2B. (TIF)

S2 Fig Time course of *AEBP1* expression in LX-2 cells grown on Matrigel-coated plates. LX-2 cells were seeded at 1 x 10^5^ cell/well on 6-well plates coated with 350 μL of (1mg/mL) Matrigel Growth Factor Reduced (GFR) basement membrane matrix (Corning Inc; Corning, NY) and cultured for 72 hours at 37°C to induce a state resembling quiescence. Transcript levels of *AEBP1* expression were normalized to *GAPDH*. All experiments were performed in triplicate. *P≤0.05.
(TIF)

S3 Fig Verification of AEBP1 knockdown efficiency in LX-2 cells. AEBP1 expression was reduced and gene expression measured as described in the Methods section. A t-test was performed to assess statistical significance. ***P = 0.0001.
(TIF)

S4 Fig Expression of miR-372-3p and miR-373-3p in activated and MDI-treated LX-2 cells. RT-qPCR was performed and miRNAs levels analyzed as described in the Methods section. Data were analyzed using a two-tailed t-test. *P<0.05.
(TIF)

S1 Table Patient demographics and clinical characteristics. (DOCX)

S2 Table Features of AEBP1 repression strength by seven miRNAs identified using predictive algorithms. (DOCX)
