# Supplementary figures and images for: AEBP1 expression increases with severity of fibrosis in NASH and is regulated by glucose, palmitate, and miR-372-3p
Source: PLoS One. 2019 Jul 12;14(7):e0219764. doi: 10.1371/journal.pone.0219764 (PMC6625715; doi:10.1371/journal.pone.0219764)

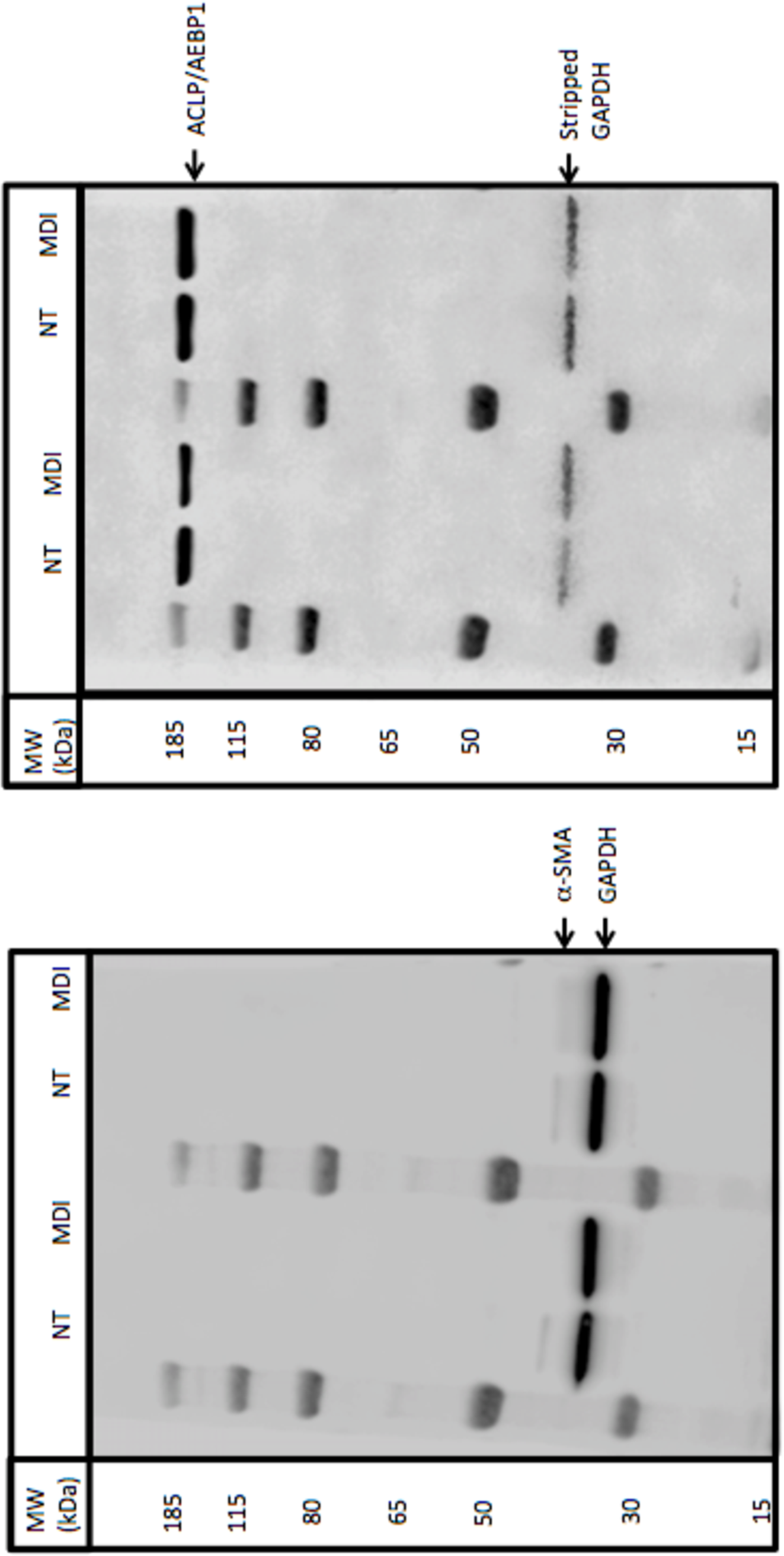

Supplement: S1 Fig — (TIF) [file pone.0219764.s001.tif]

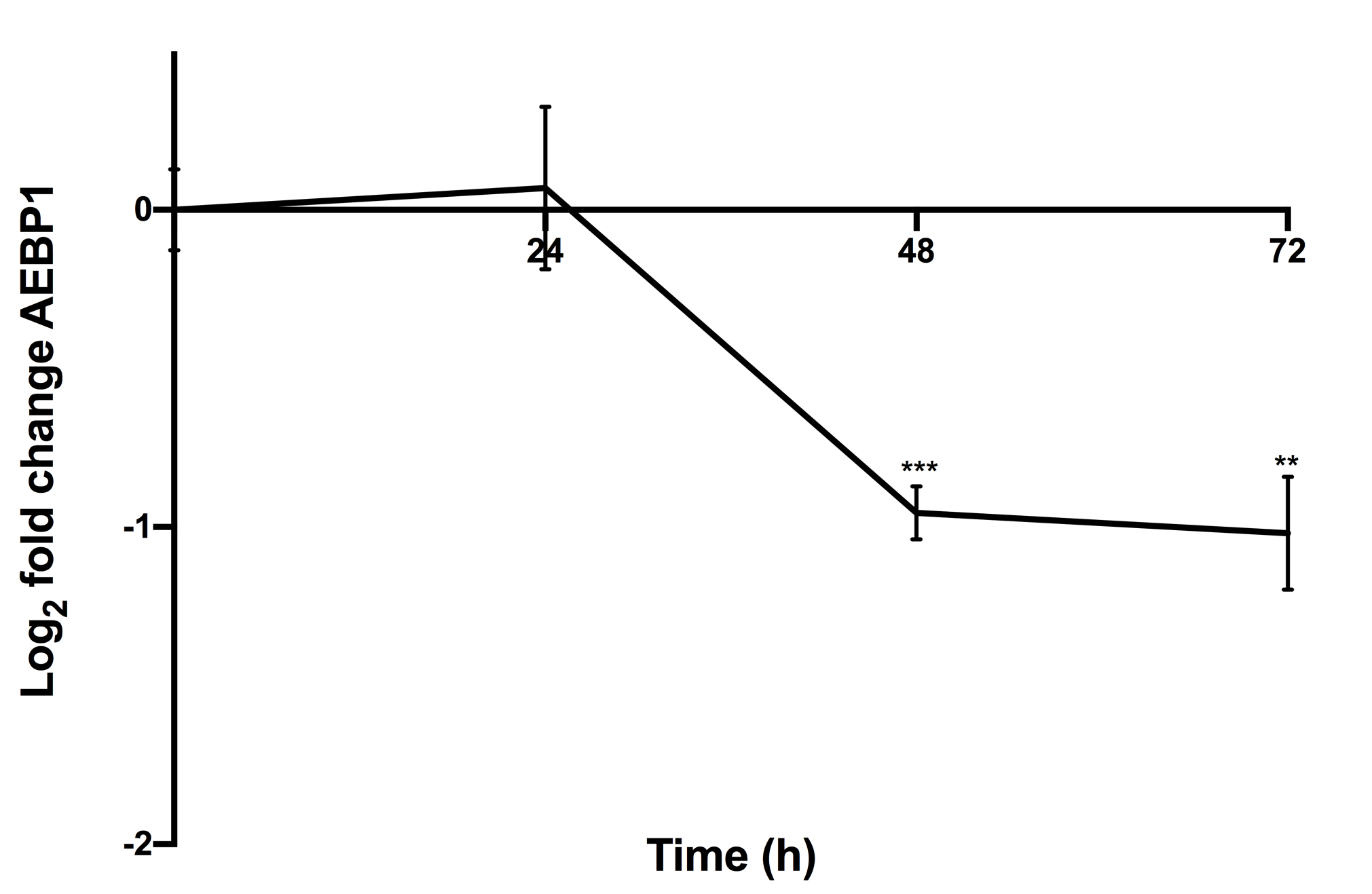

Supplement: S2 Fig — LX-2 cells were seeded at 1 x 10^5^ cell/well on 6-well plates coated with 350 μL of (1mg/mL) Matrigel Growth Factor Reduced (GFR) basement membrane matrix (Corning Inc; Corning, NY) and cultured for 72 hours at 37°C to induce a state resembling quiescence. Transcript levels of AEBP1 expression were normalized to GAPDH. All experiments were performed in triplicate. *P≤0.05. (TIF) [file pone.0219764.s002.tif]

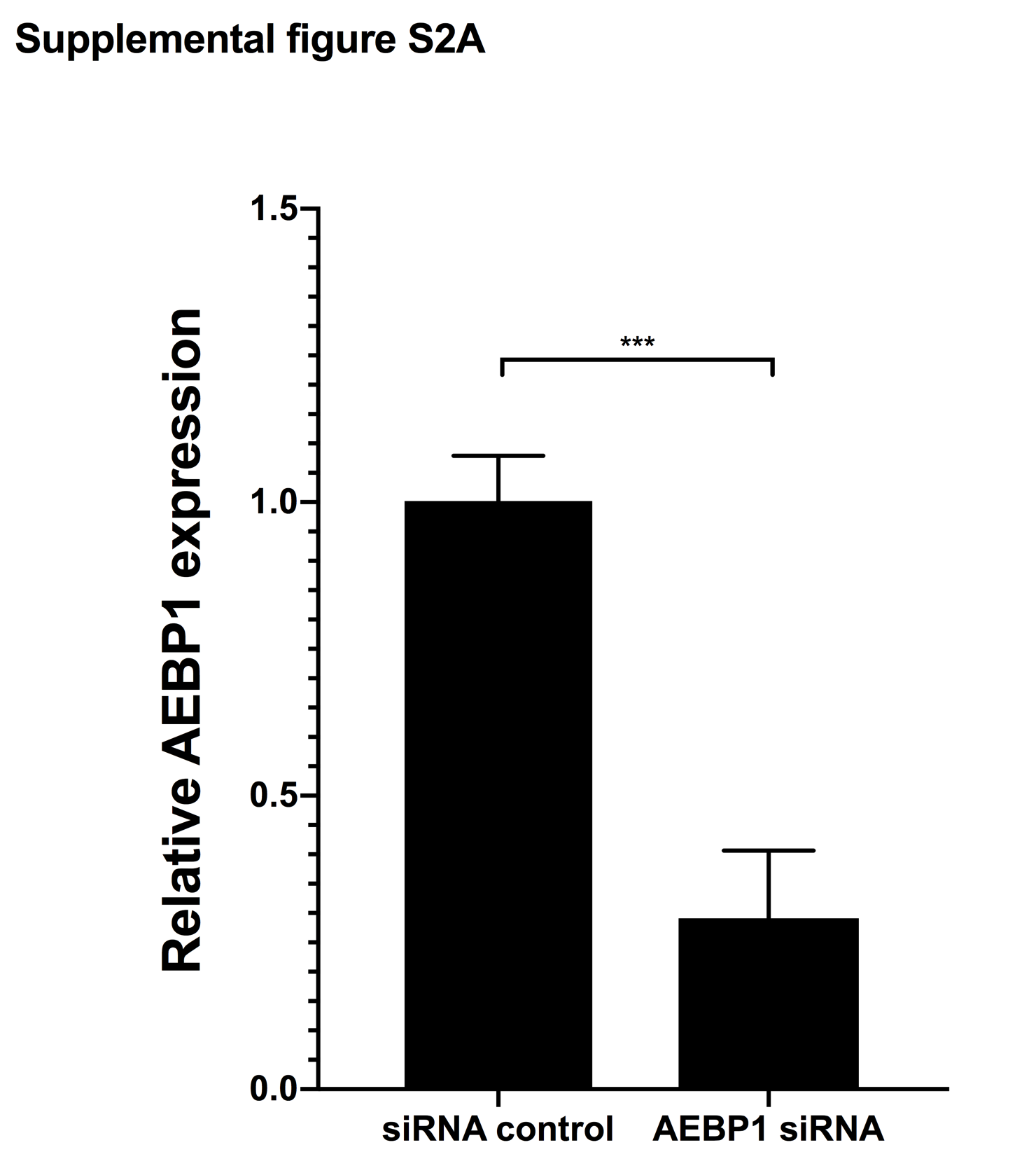

Supplement: S3 Fig — AEBP1 expression was reduced and gene expression measured as described in the Methods section. A t-test was performed to assess statistical significance. ***P = 0.0001. (TIF) [file pone.0219764.s003.tif]

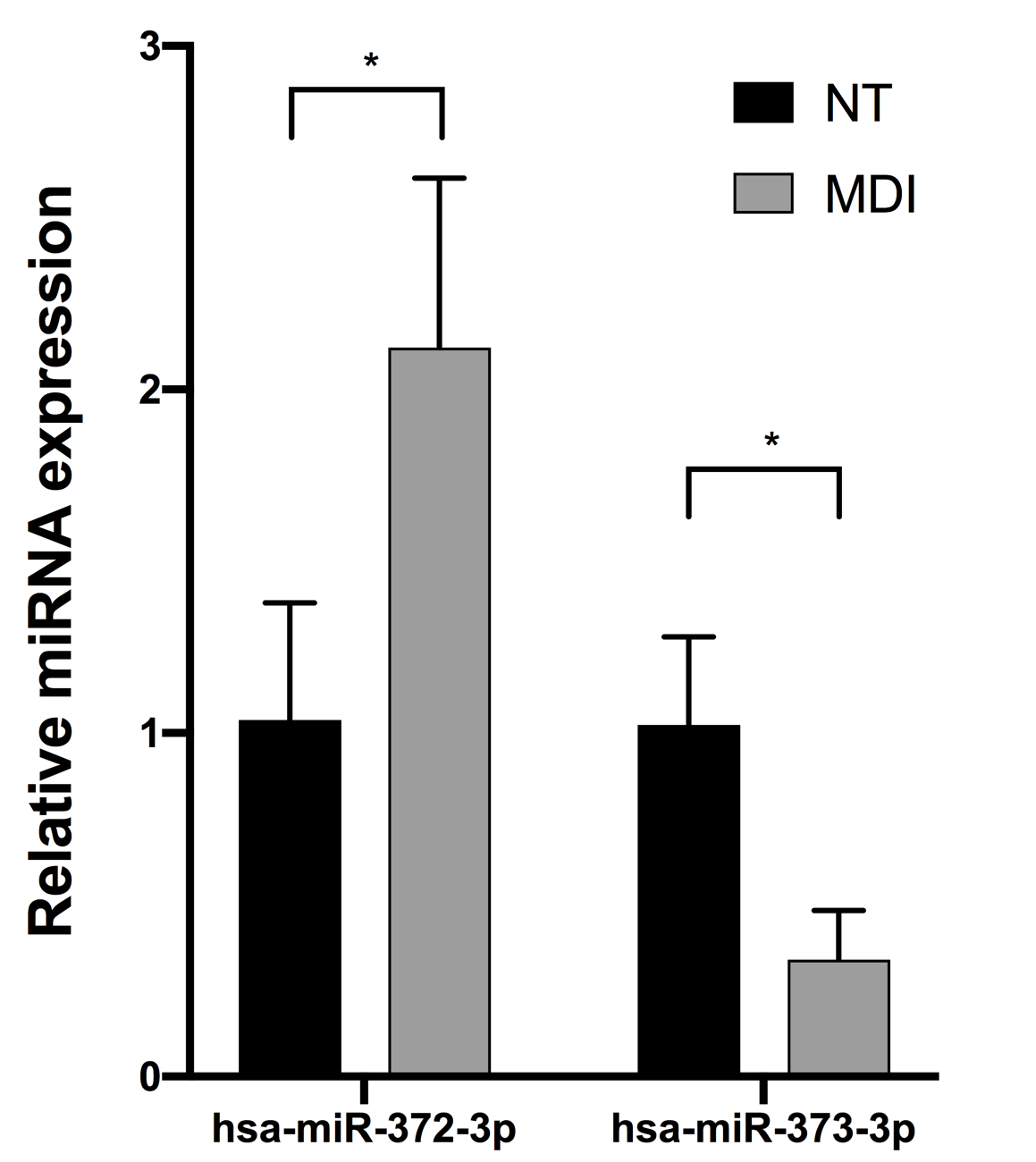

Supplement: S4 Fig — RT-qPCR was performed and miRNAs levels analyzed as described in the Methods section. Data were analyzed using a two-tailed t-test. *P<0.05. (TIF) [file pone.0219764.s004.tif]
