# Supplementary material for: AEBP1 expression increases with severity of fibrosis in NASH and is regulated by glucose, palmitate, and miR-372-3p
Source: PLoS One. 2019 Jul 12;14(7):e0219764. doi: 10.1371/journal.pone.0219764 (PMC6625715; doi:10.1371/journal.pone.0219764)
Supplement: S1 Table — (DOCX) [file pone.0219764.s005.docx]

| ***S1 Table. Patient demographics and clinical characteristics*** | | | | |
| --- | --- | --- | --- | --- |
|  | **Normal** | **Steatosis** | **Inflammation** | **Fibrosis** |
| N | 36 | 50 | 52 | 53 |
| Female, n (%) | 31 (0.86) | 39 (0.78) | 45 (0.86) | 39 (0.74) |
| Mean age in years at biopsy (SD) | 44.6 (9.8) | 46.0 (11.1) | 43.4 (12.7) | 49.1 (10.3) |
| BMI at biopsy (SD) | 43.4 (6.2) | 46.7 (9.9) | 48.8 (7.8) | 49.5 (11.1) |
| Diabetes mellitus, n (%) | 9 (0.25) | 18 (0.36) | 14 (0.27) | 36 (0.68) |
| **Laboratory measures, mean (SD)** | | | | |
| Serum AST, U/L | 21.3 (5.2) | 28.6 (8.8) | 27.9 (10.0) | 43.6 (20.2) |
| Serum ALT, U/L | 21.9 (7.9) | 37.1 (16.1) | 32.9 (15.0) | 44.3 (23.0) |
| Alkaline Phosphatase | 78.4 (16.4) | 77.2 (19.0) | 84.5 (21.2) | 87.7 (48.4) |
| Total bilirubin | 0.48 (0.2) | 0.46 (0.2) | 0.50 (0.2) | 0.57 (0.30) |
| Glucose (mg/dL) | 96.4 (26.3) | 112.5 (45.3) | 102.9 (40.2) | 134.7 (58.5) |
| Insulin | 13.0 (8.7) | 27.1 (29.2) | 25.6 (22.1) | 58.6 (73.6) |
| HbA1c, % (SD) | 5.9 (1.0) | 6.5 (1.1) | 6.5 (1.5) | 7.4 (1.6) |
| Triglycerides | 138.1 (66.3) | 165.7 (87.9) | 187.5 (91.8) | 209.6 (113.3) |
| Total cholesterol | 186.9 (34.7) | 187.0 (37.9) | 194.6 (31.8) | 184.9 (39.5) |
| LDL-C | 106.6 (30.8) | 109.9 (40.0) | 108.2 (30.9) | 101.9 (34.8) |
| HDL-C | 53.0 (11.2) | 47.2 (9.7) | 49.0 (11.0) | 42.2 (12.1) |
|  | | | | |
