# Supplementary material for: AEBP1 expression increases with severity of fibrosis in NASH and is regulated by glucose, palmitate, and miR-372-3p
Source: PLoS One. 2019 Jul 12;14(7):e0219764. doi: 10.1371/journal.pone.0219764 (PMC6625715; doi:10.1371/journal.pone.0219764)
Supplement: S2 Table — (DOCX) [file pone.0219764.s006.docx]

| **S2 Table. Features of AEBP1 repression strength by seven miRNAs identified using predictive algorithms** | | | | |
| --- | --- | --- | --- | --- |
| **ID** | **ΔG open^1^** | **Probability^2^** | **Conservation^3^** | **miRmap score^4^** |
| hsa-mir-134-5p | 55.46 | 81.40 | 0.62 | 77.46 |
| hsa-mir-30e-3p | 27.58 | 91.86 | 0.62 | 35.07 |
| hsa-mir-512-3p | 43.51 | 73.94 | 0.62 | 79.42 |
| hsa-mir-1262 | 39.09 | 82.24 | 0.62 | 88.30 |
| hsa-mir-3619-5p | 68.13 | 71.25 | 0.62 | 85.03 |
| hsa-mir-372-3p | 43.48 | 93.06 | 0.62 | 77.63 |
| hsa-mir-373-3p | 43.48 | 93.06 | 0.62 | 72.40 |
| *^1^mRNA opening free energy-Accessibility*  *^2^Site over-representation probability (exact distribution)*  *^3^determined using the SPH test from phyloP*  *^4^miRNA repression strength* | | | | |
